# ESTree db: a Tool for Peach Functional Genomics

**DOI:** 10.1186/1471-2105-6-S4-S16

**Published:** 2005-12-01

**Authors:** Barbara Lazzari, Andrea Caprera, Alberto Vecchietti, Alessandra Stella, Luciano Milanesi, Carlo Pozzi

**Affiliations:** 1 Parco Tecnologico Padano, Via Einstein – Località Cascina Codazza, 26900 Lodi, Italy; 2 CISI, Via Fratelli Cervi 93, 20090 Segrate (MI), Italy; 3 Istituto Tecnologie Biomediche, Via Fratelli Cervi 93, 20090 Segrate (MI), Italy

## Abstract

**Background:**

The ESTree db  represents a collection of *Prunus persica *expressed sequenced tags (ESTs) and is intended as a resource for peach functional genomics. A total of 6,155 successful EST sequences were obtained from four in-house prepared cDNA libraries from *Prunus persica *mesocarps at different developmental stages. Another 12,475 peach EST sequences were downloaded from public databases and added to the ESTree db. An automated pipeline was prepared to process EST sequences using public software integrated by in-house developed Perl scripts and data were collected in a MySQL database. A php-based web interface was developed to query the database.

**Results:**

The ESTree db version as of April 2005 encompasses 18,630 sequences representing eight libraries. Contig assembly was performed with CAP3. Putative single nucleotide polymorphism (SNP) detection was performed with the AutoSNP program and a search engine was implemented to retrieve results. All the sequences and all the contig consensus sequences were annotated both with blastx against the GenBank nr db and with GOblet against the viridiplantae section of the Gene Ontology db. Links to NiceZyme (Expasy) and to the KEGG metabolic pathways were provided. A local BLAST utility is available. A text search utility allows querying and browsing the database. Statistics were provided on Gene Ontology occurrences to assign sequences to Gene Ontology categories.

**Conclusion:**

The resulting database is a comprehensive resource of data and links related to peach EST sequences. The Sequence Report and Contig Report pages work as the web interface core structures, giving quick access to data related to each sequence/contig.

## Background

The development of the ESTree db  is one of the primary objectives of the ESTree Interuniversitary Centre, which is clustering several research units in Italy, devoted to the implementation of genomics and functional genomics in *drupaceae *species [1].

In the frame of the activities of this centre, four cDNA libraries from *Prunus persica *mesocarps from three different cultivars (cv) at different developmental stages (post-fertilization, endocarp hardening, pre-climateric and post-climateric final maturation steps) were prepared and named S1 (cv Suncrest), S2 (cv Bolero), S3 (cv OroA) and S4 (cv Bolero), respectively (unpublished results). For each library, extensive EST sequencing was performed and a total of 6,155 successful sequences was obtained and submitted to the EBI databank for assignment of GenBank accession numbers. Sequences were given the accession numbers from AJ633783 to AJ633817, from AJ821924 to AJ827750 and from AJ870494 to AJ870786. Another 12,475 peach EST sequences from four libraries representing different fruit developmental stages and different cultivars (Loring, Fantasia and Redhaven) were obtained from public databases and added to our sequences, so that a dataset of 18,630 peach EST sequences was eventually created. Sequence data interchange was established with Dr. Albert Abbott's lab at Clemson University, and thus the ESTree db and the GDR rosaceae db [2] partially share the same sequences.

The aim of this work was to produce an extensive and easily accessible EST database for peach with links to other related sources. The ESTree db is intended to be the repository of all data produced by the ESTree Interuniversitary Centre members, and has to be considered as a dynamic structure where new sequences and new features will continuously be added. The other main web data resource on peach EST sequences is the GDR rosaceae database: despite a partial redundancy in data representation, each database is featuring some different element. Putative SNP detection as well as links to external sources, such as the NiceZyme enzyme database and the KEGG pathways database, are specific to the ESTree db. Furthermore, in the ESTree db the text search utility is active on a larger number of fields than in the GDR db and a more detailed alignment of EST contigs is provided.

An additional database (ESTree_quality: ) was produced to store only sequences provided along with correlated quality files.

## Construction and content

### The Pipeline

**Figure 1:**
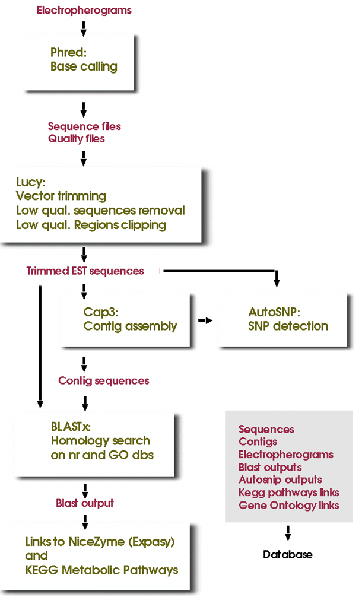
**The ESTree db pipeline**. Data flow in the ESTree db pipeline. Accessory in-house developed Perl programs are not shown in the scheme.

An automated pipeline (Fig. 1) was prepared to process EST sequences using public software integrated by a number of in-house developed Perl scripts.

From extensive sequencing of the S1, S2, S3 and S4 libraries a total of 7,161 sequences was obtained. Sequencing was mostly performed on libraries S3 and S4, as preliminary library quality controls indicated a higher mean insert length in comparison to S1 and S2 libraries. The 7,161 electropherograms were read with the program Phred [3] and multifasta sequence and quality files were created. These files were parsed and stored in a MySQL database, together with the electropherograms and data retrieved during the various steps of the pipeline. Multifasta files were fed into the program Lucy [4] and both low quality regions and vector sequences were removed, according to Lucy standard parameters, leading to 6,155 successful sequences longer than 100 bp. As 3' sequencing was performed, vector free, high quality sequences were reverse-complemented with the program REVSEQ (EMBOSS). The file containing the forward sequences was appended to the file containing the 12,475 peach EST sequences downloaded from GenBank, and a single multifasta file containing the complete peach EST dataset was created. Full details on the source of downloaded sequences and library of origin are included in the Home page and in the Library details page of the cited web site.

The complete dataset was used as input for the CAP3 program [5]. CAP3 parameters were set to -p 98, -o 100, for appropriate EST clustering. The CAP3 output was parsed and relevant data were stored in the database. An unigene dataset was defined, including all singlet sequences and the longest sequence for each contig.

In order to retrieve putative *in silico *single nucleotide polymorphisms (SNPs) the complete peach EST dataset was also fed into the AutoSNP program [6]. AutoSNP invokes TGICL [7] for sequence clustering and CAP3 for contig assembly. TGICL parameters were set to -p 95, -l 60 and -v 20 and CAP 3 parameters were set to -p 96 and -o 100.

All the 18,630 EST sequences and all the contig consensus sequences were annotated with a double procedure. The first annotation was performed locally with blastx [8], versus the nr database downloaded from GenBank (referred to as "NCBI blast" in the web interface). For this purpose, the mpiBLAST [9] program was used, allowing parallelization of the Blast procedure. Blast was performed on a Linux 9-biprocessor AMDx64 cluster with 2 GB RAM on each node. 54 blast outputs per minute were retrieved, allowing the entire sequence annotation process to be completed in less than six hours. Low complexity filters were disabled, while the remaining blastx parameters were the defaults and no threshold was set. The BLAST output was parsed with an in-house prepared parser. The most important BLAST output values, as well as the complete BLAST output pages, were stored in the database.

The second annotation was performed online with GOblet [10] versus the viridiplantae subset of the GO database [11] (a subset of sp-trembl; referred to as "GO blast" in the cited web interface). GOblet annotation is obtained by blastx; in this case the E-value threshold of 1e-10 was adopted. GOblet produced three output files for each input sequence. Ontologies were derived from the GOblet output files and stored in the database, to allow successive dynamic creation of statistics for the ontology occurrences.

The NCBI blast output files were scrolled for the presence of EC numbers. When present in the Best Blast Hit description line, these were retrieved and used to search for correspondent links in the Enzyme nomenclature database (Expasy) [12] and in the related NiceZyme interface. Links to the KEGG Pathways database [13] were also derived from the EC numbers and retrieved. NiceZyme enzyme descriptions and KEGG links were stored in the database. Further details on software usage and parameters settings are provided at the Processing, Assembly and Annotation Protocol page of the cited web site.

Statistics on the sequence analysis are presented in Table 1.

### The Database

Currently, the ESTree MySQL database consists of 15 main tables. An extra table is added for each library-specific GO statistic. As the ESTree db is constantly growing and new features are being added, the database structure is subject to modifications.

**Figure 2:**
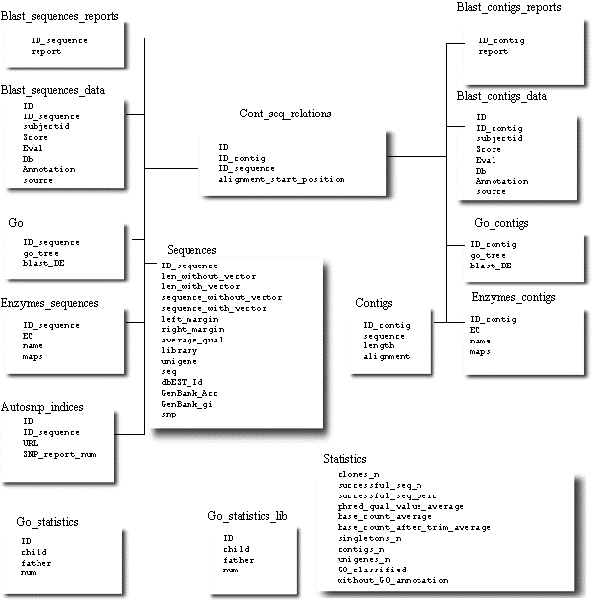
**The ESTree db database structure**. Main tables of the ESTree MySQL db. The database structure is subject to frequent changes, due to the implementation of the database features.

The "Sequences" table and the "Contigs" table are the core structures of the database, together with the "Cont_Seq_relations" table, where relations among sequences and contigs are defined (Fig. 2).

### The Web Interface

**Figure 3:**
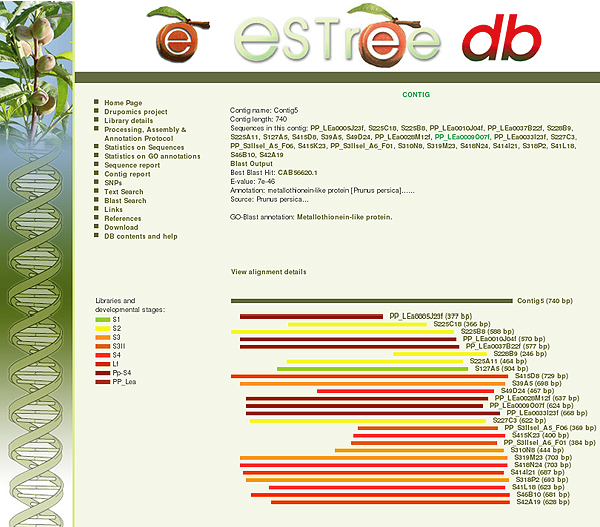
**The ESTree db Contig display page**. An example of the ESTree db contig graphical display. The bar colours reflect the developmental stage of the sequence clone library of origin. The same colours recur in the AutoSNP output and in the library details. The graphical display is dynamically created by the php web interface.

The ESTree db web interface is based on the php language and manages all the incoming queries as well as all the graphically-presented data dynamic output creation. Contig graphical display and GO statistics bars are prepared on-the-fly in response to the users request and are not stored in any part of the database (Fig. 3).

## Utility and discussion

### The ESTree db Structure

In virtue of its flexibility, the ESTree db pipeline was also used in EST analyses for related projects, with different input datasets [14]. Data flow was maintained through the entire process, but allowing the preparation of dataset-specific outputs. The contig assembly process was kept apart from the putative SNP detection procedure, allowing the two processes to be carried out independently. In some cases, different features were added and easily integrated in the procedure; i.e. blast analysis versus species specific genomic sequences (unpublished results).

Part of the data displayed in the ESTree site is produced on-the-fly by the php-based web interface. This is mostly referred to graphical outputs. In particular, statistics on Gene Ontology occurrences would require updating each time the GO blast is performed, as well as contig graphical display would require being updated each time new sequences are added to the database. The dynamic management of all these data provides always up-to-date displays without the need for refreshing corresponding database tables.

### The ESTree db web Site

The ESTree db web site was designed to allow easy data retrieval.

The Sequence report and Contig report pages are the starting points for sequence and contig related data access. Data in these pages are presented in summary tables and are extensively linked to external sources. Furthermore, each sequence and each contig were assigned a detailed page where the nucleotide sequence can be copied. In each contig page, the contig graphical display is provided, as well as the contig alignment. NCBI blast outputs are accessible in the original format for each sequence and each contig consensus sequence, reporting the description of the best ten hits and the alignments of the best five hits. GO blast original outputs are also retrievable.

Statistics were provided both on the sequence dataset status and on Gene Ontology occurrences. GO statistics were prepared for the whole set of sequences and for library-specific subsets and both hierarchical and non-hierarchical browsing of categories is allowed.

A text search utility was implemented to search sequence data and contig data. As NCBI blast results are presented independently of their significance, a user-defined significance threshold (E-value) can be set in the search to retrieve only significantly annotated sequences. Searches can be restricted to database subsets (unigenes/not unigenes, singlets/contig related sequences, putative SNP containing sequences). Query outputs can be downloaded both as multifasta sequence files and as text files with tab separated values, containing all the values for the sequence/contig report page fields. These can be easily imported in Excel or other spreadsheets.

Local blasting of user's sequences against the nucleic ESTree db and the protein database containing the translations of the EST sequences is allowed. Blast can be restricted to the subset of putative SNP-containing sequences.

Links to the NiceZyme (Expasy) database and to the KEGG metabolic pathways tables were added allowing simultaneous retrieval of data from different data sources. As via the text search interface searches can be restricted to sequences/contigs with an associated NiceZyme or KEGG entry, a quick view on the bulk of peach enzyme sequences that are present in the ESTree db can be easily obtained.

From the Download page, download of the ESTree sequences is allowed in multifasta, CSV and GenBank formats. Contig consensus sequences can be downloaded in multifasta and CSV formats. AutoSNP SNP reports can also be downloaded in html format.

The database includes a detailed help page to assist users in browsing the ESTree db and in interpreting the outputs.

Previous releases of the ESTree db are maintained and links to these versions are given in the home page. This is intended to allow frequent users to retrieve old contig assemblies. Sequence annotations will be periodically updated, and the consistency of external links is constantly verified.

### Future developments

Among the primary objectives of the ESTree Interuniversitary Centre is the microarray analysis on peach. A significant number of 70 mer oligonucleotides was synthesized, based on the peach EST unigene dataset, and microarrays are under test (unpublished results). Data obtained from microarray analysis will be included in the ESTree db.

Blastn analysis results for the whole set of EST sequences and contigs against the genomic *rosaceae *sequences will be included.

SNP-mapping of ESTs on available molecular linkage maps is also underway (unpublished results) and related data will be included in the ESTree db.

The pipeline will be modified in order to avoid discrepancies in assemblies between contigs generated with CAP3 and with AutoSNP.

An alternative putative SNP detection analysis will be reported in the ESTree_quality web database, performed with sequence quality based algorithms.

## Conclusion

The ESTree db version as of April 2005 encompasses 18,630 sequences obtained from eight libraries, but the database is continuously growing and new features are being added.

The ESTree db proved to be very useful for the selection of sequences to be used for specific purposes (i.e. SNP-mapping). In particular, the possibility to partition the dataset and perform subset-specific text searches offers an efficient tool for data mining and data retrieval.

The ESTree db is the first web resource reporting data on putative SNP sites in peach, and will be the main repository of data obtained by the ESTree Interuniversitary Centre units, allowing the creation of a platform for easy data integration and retrieval, with the aim to provide a tool to improve knowledge on peach genomics and functional genomics.

## Availability and requirements

The ESTree db is available at .

The derivative ESTree_quality db is available at .

## Authors' contributions

BL participated to library preparation, defined the pipeline structure and parameters and drafted the manuscript. AC structured the database and wrote all the accessory programs of the whole project. AV was responsible for library preparation and sequence management. AS participated in the design of the study and critically revised the manuscript. LM coordinated the integration of bioinformatical resources. CP guided and coordinated the execution of the project and was the main source of motivation for building the ESTree db. All authors read and approved the final manuscript.

## Figures and Tables

**Table 1:** Statistics on sequence analysis. Data are derived from the outputs of the programs invoked by the pipeline. ^(1)^Each SNP report contains data on one or more putative SNP sites. ^(2)^The unigene dataset encompasses all the singlets plus the longest sequence of each contig. ^(3)^Annotation threshold: E-value < 1e-10.

	Sequence Number	Sequence %
Total number of sequences	18,630	
Average sequence base count	544.52	
Number of singletons	6,891	36.99
Number of contigs	2,328	
Number of sequences in contigs	11,739	63.01
Average number of sequences in each contig	5.04	
Number of SNP reports ^(1)^	166	
Number of putative unigenes ^(2)^	9,219	49.48
Annotated sequences (NCBI blast) ^(3)^	13,114	70.39
Annotated sequences (GO blast) ^(3)^	9,056	48.61
Number of enzyme sequences	661	3.55
Number of sequences linked to KEGG metabolic pathways	282	1.51
